# Peripheral transcriptomic aging acceleration in major depressive disorder: the mediating role of insular cortex alterations

**DOI:** 10.1017/S003329172610498X

**Published:** 2026-07-08

**Authors:** Yushun Yan, Min Wang, Yuanmei Tao, Jinxue Wei, Liansheng Zhao, Rongjun Ni, Xiao Yang, Xiaohong Ma

**Affiliations:** Mental Health Center and Institute of Psychiatry, National Center for Mental Disorders, West China Hospital, Sichuan University, Chengdu, China

**Keywords:** biological aging acceleration, insular cortex, major depressive disorder, peripheral transcriptome

## Abstract

**Background:**

Biological aging may contribute to the pathogenesis of major depressive disorder (MDD). However, whether and how peripheral transcriptomic aging increases the risk of MDD onset remains unclear.

**Methods:**

Transcriptomic age was estimated using peripheral blood RNA sequencing data from 141 individuals with MDD and 134 healthy controls. The residuals of transcriptomic age regressed on chronological age were calculated to indicate transcriptomic aging acceleration. Enrichment analysis was performed to explore potential biological mechanisms underlying aging- and MDD-associated transcriptomic alterations. Associations between transcriptomic aging and clinical, neurocognitive, environmental, genetic, and neuroimaging phenotypes were examined.

**Results:**

Participants with MDD exhibited significantly accelerated transcriptomic aging both before (*t* = 2.06, *P* = 0.040) and after adjusting for chronological age and sex (*t* = 3.72, *P* < 0.001). Enrichment analysis revealed shared terms in innate immune-related inflammation, ribosome biogenesis, and mitochondrial energy metabolism, while telomere length maintenance was specifically enriched in aging but not in MDD. No significant associations were found between transcriptomic aging and clinical symptoms, neurocognitive functions, childhood trauma exposure, or polygenic risk score. Neuroimaging analyses demonstrated that transcriptomic aging was associated with structural (*t* = −3.30, *P* = 0.001) and functional (*t* = 2.64, *P* = 0.009) alterations in the right insular cortex. Further analyses indicated that insular abnormalities partially mediated the impact of transcriptomic aging on MDD vulnerability.

**Conclusions:**

Transcriptomic aging may represent a novel risk factor for MDD. Disruption in the insular cortex may serve as a critical neural substrate through which accelerated transcriptomic aging increases vulnerability to MDD.

## Introduction

The pathogenesis of major depressive disorder (MDD) involves both biological and environmental factors, such as genetic predispositions, transcriptomic alterations, and early life stress (Marx et al., 2023). Aging is a lifelong process driven by both programmed genetic expression and the cumulative damage experienced by individuals across the lifespan, which is similarly influenced by biological and environmental contributors (Moqri et al., 2023). Both MDD and aging are associated with increased risks of age-related conditions, including chronic disease (Berk et al., 2023), frailty (Deng et al., 2023), and cognitive decline (Semkovska et al., 2019). MDD encompasses biological processes commonly observed in aging, including mitochondrial dysfunction (Picard & McEwen, 2018), telomere shortening (Mendes-Silva et al., 2021), chronic inflammation (Ishizuka, Nagata, Nakagawa, & Takahashi, 2024), and immune dysregulation (Diniz et al., 2022). These similarities have led to the hypothesis that aging-related processes are associated with the pathophysiology of MDD.

To capture the progressive accumulation of molecular, cellular, and tissue damage during the aging process, various biological aging clocks have been developed. Such models based on epigenomic (Duan, Fu, Sun, & Li, 2022), metabolomic (Zhang et al., 2024), and proteomic (Tanaka et al., 2020) markers have revealed evidence of significant deviations between biological and chronological age. As a downstream layer of the genome, the transcriptome reflects both intrinsic genetic programs and extrinsic regulatory influences, shaped by mechanisms such as DNA methylation, histone modification, and chromatin architecture remodeling, all of which undergo dynamic changes across the lifespan (Meyer & Schumacher, 2021). Therefore, the transcriptome holds the potential as a biomarker of aging (Peters et al., 2015). Notably, transcriptomic alterations in peripheral blood have also been reported in individuals with MDD, raising the possibility that MDD may be associated with age-related changes in transcriptomic profiles (Ciobanu et al., 2016). Nevertheless, existing literature does not elucidate whether MDD is associated with accelerated transcriptomic aging, or how aging-related transcriptomic alterations may contribute to the onset of MDD.

In the current study, we aimed to investigate whether MDD exhibits signs of accelerated biological aging as reflected by peripheral transcriptomic markers. Furthermore, we examined the extent to which transcriptomic aging accounts for variability in clinical symptoms, neurocognitive performance, childhood trauma scores, polygenic risk scores, and neuroimaging features. We hypothesized that transcriptomic aging is accelerated in MDD and serves as an important vulnerability factor contributing to increased susceptibility to MDD.

## Methods

### Participants

**Figure 1.**
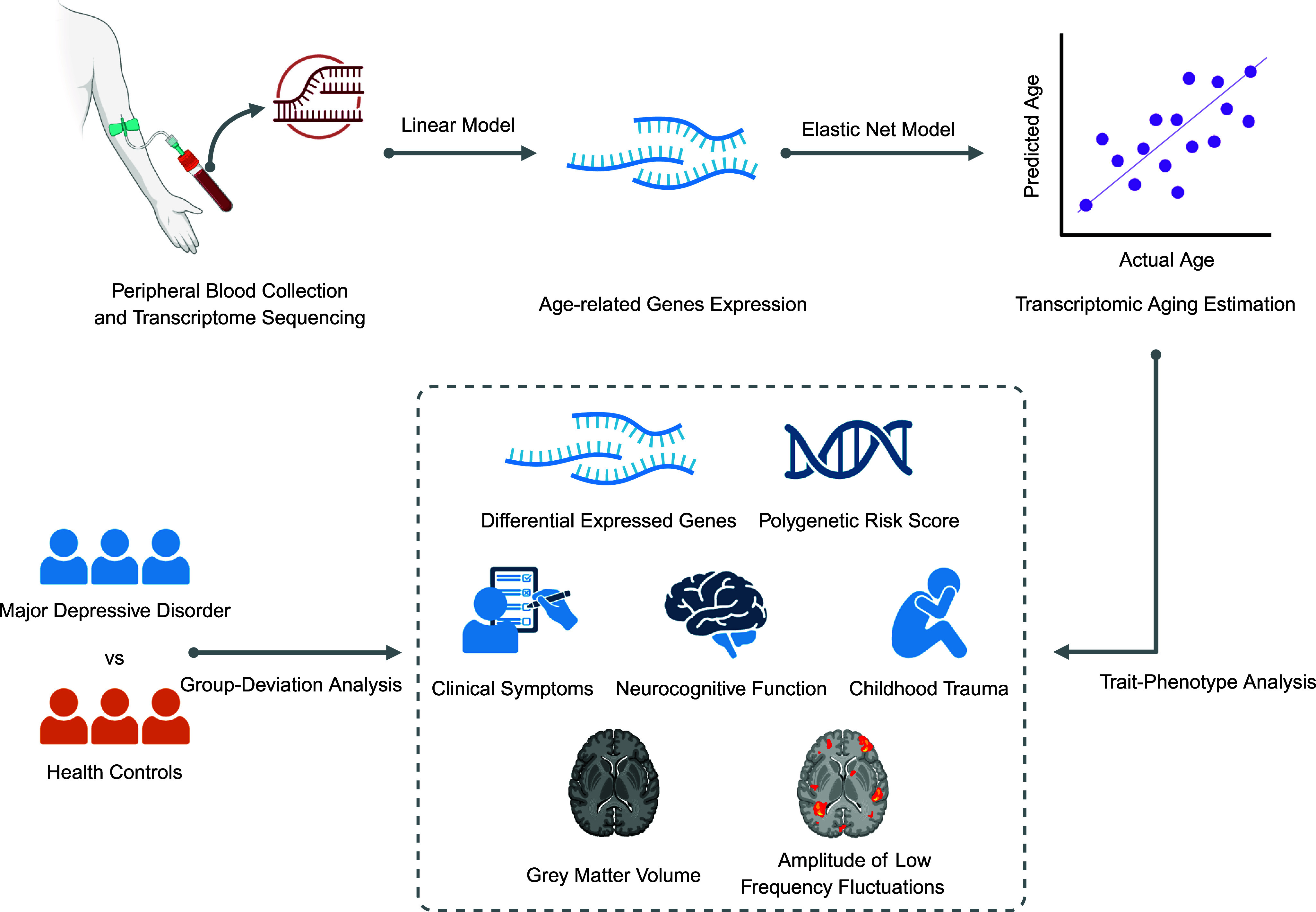
Overview of study design and analytical framework. *Note*: Peripheral blood samples were collected for transcriptome sequencing to obtain gene expression profiles. Age-related genes were identified using linear regression models, followed by the construction of an elastic net regression model to predict transcriptomic age. The difference between predicted transcriptomic age and actual chronological age was used to estimate transcriptomic aging. Further analyses were performed to examine group deviations and aging associations in various phenotypes, including differentially expressed genes, polygenic risk scores, clinical symptoms, neurocognitive function, childhood trauma, gray matter volume, and amplitude of low-frequency fluctuations. Figure 1. long description.

Participants diagnosed with MDD were recruited from the Mental Health Center of West China Hospital, Sichuan University. Diagnosis was established according to the criteria of the Diagnostic and Statistical Manual of Mental Disorders, Fifth Edition (DSM-5), based on independent clinical evaluations conducted by two psychiatrists. Eligible participants were of Han Chinese ethnicity, aged 16–40 years, and were either antidepressant-naïve or had discontinued antidepressant medication for a minimum of 3 months prior to enrollment. Exclusion criteria included any comorbid psychiatric disorders (e.g., schizophrenia, bipolar disorder), neurological or systemic somatic diseases (e.g., traumatic brain injury, endocrinology, metabolism, or autoimmune disorders), pregnancy or lactation, color vision deficiencies, or any contraindications to magnetic resonance imaging (MRI). Healthy controls (HCs) were recruited through public advertisements and were included only if they, as well as their first-degree relatives, had no history of psychiatric disorders. They were further evaluated through structured interviews to confirm the absence of any current or past psychiatric disorders, physical illnesses, a family history of psychiatric disorders in first-degree relatives, and any use of medications that could potentially affect the central nervous system within the past 6 months. Written informed consent was obtained from all participants and/or their legal guardians. The study protocol was approved by the Ethics Committee of West China Hospital, Sichuan University (No. [2016]170) and conducted in accordance with the Declaration of Helsinki. The overall study workflow is illustrated in Figure 1.

### Phenotypes assessments

Information on chronological age, sex, depressive episode history, suicidal ideation and behaviors was obtained through clinical interviews. The severity of depression was measured using the Hamilton Depression Rating Scale (HDRS) (Hamilton, 1960), and co-occurring anxious distress was evaluated using the Hamilton Anxiety Rating Scale (HARS) (Hamilton, 1959). Neurocognitive function was assessed using the Cambridge Neuropsychological Test Automated Battery (CANTAB), which included six subtests: delayed matching to sample (DMS) for visual memory, pattern recognition memory (PRM) for recognition memory, rapid visual information processing (RVP) for sustained attention, intra–extra dimensional set shift (IED) for cognitive flexibility, stockings of Cambridge (SOC) for planning and problem solving, and spatial working memory (SWM) for working memory.

### Peripheral transcriptomic analysis

#### mRNA collection, extraction, and sequencing

Peripheral blood samples were obtained from participants on the day of enrollment. Total RNA was extracted using the MagMAX for Stabilized Blood Tubes RNA Isolation Kit (Thermo Fisher Scientific, Waltham, MA, USA) following the manufacturer’s protocol. RNA sequencing was conducted at GENEWIZ Suzhou (Suzhou Genewiz Biotechnology Co., Ltd., Suzhou, China) using the Illumina NovaSeq 6000 platform with a 2 × 150 bp paired-end configuration and standard mRNA-seq protocols (Supplementary Method 1). All RNA samples were sequenced simultaneously in a single batch.

#### Transcriptomic data processing

Gene-level read counts, derived from aligned and filtered clean reads, were processed. Gene-level count data were filtered to retain only those with counts per million (CPM) > 1 in at least 90% of samples within either group. Library sizes were recalculated, and normalization was performed using the trimmed mean of *M*-values method to account for library composition differences (*edgeR* package). To identify age-associated genes, we adopted an approach consistent with previous studies by fitting linear models for each gene, with log-transformed CPMs as the outcome and chronological age, sex, and MDD diagnosis as the predictors (Peters et al., 2015). *P*-values obtained from the models were adjusted for multiple comparisons using the Benjamini–Hochberg false discovery rate method. Genes with an adjusted *P*-value for age lower than 0.05 were considered significantly associated with chronological age, whereas genes with an adjusted *P*-value for group differences lower than 0.05 were considered differentially expressed between MDD and HC.

#### Transcriptomic aging estimation

To obtain unbiased estimates of transcriptomic age in our sample, we adopted an approach consistent with previous studies (Han et al., 2018; Hwangbo et al., 2022). Specifically, we used the significantly age-associated genes as predictors in an elastic net regression model, with chronological age as the outcome variable (*glmnet* package). Model fitting and parameter selection were performed using 10-fold cross-validation. The predicted values from the best-fitting model were taken as transcriptomic age estimates. Transcriptomic aging was calculated as follows:

$$\text{Transcriptomic Aging}=\frac{\text{Predicted}\;\mathrm{Age}-\text{Chronological}\;\mathrm{Age}}{\text{Chronological}\;\mathrm{Age}}$$

#### Gene ontology enrichment analysis

Gene set enrichment analysis was performed using Gene Ontology (GO) biological process and Kyoto Encyclopedia of Genes and Genomes (KEGG) pathway databases to explore the functional relevance of age-associated and MDD-associated genes, respectively (*clusterProfiler* package). All genes were ranked according to the *t* statistics derived from the linear models, thereby avoiding predefined thresholds for gene selection. Multiple testing correction was performed using the Benjamini–Hochberg procedure. Gene sets with an adjusted *P*-value <0.01 were considered significantly enriched, and results were ordered by the absolute normalized enrichment score (NES).

### Psychosocial factor assessment

Childhood trauma scores were derived from the Childhood Trauma Questionnaire (CTQ) (Bernstein et al., 2003), a retrospective self-report scale consisting of 28 items rated on a 5-point Likert scale. Three items (Items 10, 16, and 22) were used for validity assessment and were excluded from total score calculation. Reverse coding was applied to the relevant items (Items 2, 5, 7, 13, 19, 26, and 28) in accordance with standard scoring procedures. Total scores ranged from 25 to 75, with higher scores indicating greater severity of childhood trauma. The CTQ has been validated in Chinese populations, including individuals with MDD, demonstrating acceptable internal consistency (Cronbach’s *α* = 0.77; He et al., 2019). Previous studies have also suggested that retrospective assessment of childhood trauma using the CTQ is relatively stable and not substantially influenced by current mental state (Bernstein et al., 1994; Pinto, Correia, & Maia, 2014).

### Polygenic risk score calculation

Genomic DNA was extracted from peripheral blood cells using the phenol–chloroform protocol. Genotyping was performed at Meiji Gene (Shanghai Biowing Applied Biotechnology Co., Ltd., Shanghai, China) using the Illumina Global Screening Array-24+ v1.0 BeadChip on the Illumina iScan System, following the manufacturer’s recommended protocol. After quality control, 242,681 variants remained for downstream analysis (Supplementary Method 2). All DNA samples were sequenced simultaneously in a single batch.

Polygenic risk scores were calculated using *PRSice-2* with genotype data processed in *PLINK* format. Summary statistics were obtained from a large-scale genome-wide association study of MDD conducted in East Asian populations, excluding participants from 23andMe and UK Biobank to avoid sample overlap (Giannakopoulou et al., 2021). To ensure high-quality variant inclusion, single nucleotide polymorphisms with minor allele frequency <0.01 or imputation INFO score < 0.8 were excluded from the summary statistics. Linkage disequilibrium (LD) reference panels were based on East Asian samples from Phase 3 of the 1000 Genomes Project. In the target dataset, the first five principal components were extracted and included as covariates to control for population stratification. SNPs were clumped based on LD and selected using a *P*-value threshold of 5 × 10^−5^ < *P* < 5 × 10^−8^. The final model was determined by identifying the *P*-value threshold that explained the largest proportion of phenotypic variance (maximum R^2^) in the sample.

### Neuroimaging analysis

#### Magnetic resonance images acquisition and preprocessing

MRI data were acquired using a Philips 3T scanner (Philips, Amsterdam, the Netherlands) equipped with an eight-channel phased-array head coil.

High-resolution T1-weighted structural images were collected using the following parameters: echo time (TE) = 3.88 ms, repetition time (TR) = 8.4 ms, flip angle = 7°, acquisition matrix = 256 × 256, field of view (FOV) = 240 × 240 mm^2^, slice thickness = 1 mm, voxel size = 1 × 1 × 1 mm^3^, and number of slices = 188. Preprocessing procedure followed the default pipeline of the computational anatomy toolbox (*CAT12*) implemented in *SPM12* (Supplementary Method 3) (Gaser et al., 2024). Gray matter volume (GMV) maps of whole-brain voxels were obtained.

Resting-state functional images were acquired with the following parameters: TE = 30 ms, TR = 2000 ms, flip angle = 90°, acquisition matrix = 64 × 64, FOV = 240 × 240 mm^2^, slice thickness = 4 mm, voxel size = 3.75 × 3.75 × 4 mm^3^, number of slices = 38, and number of time points = 240. Participants were instructed to remain still and awake with their eyes closed during the scanning procedure. Preprocessing and first-level analysis procedures used the default *fMRIPrep* pipeline (Esteban et al., 2019) and *CONN* toolbox (Whitfield-Gabrieli & Nieto-Castanon, 2012), respectively (Supplementary Method 4). Amplitude of low-frequency fluctuations (ALFF) maps of whole-brain voxels were obtained.

#### Voxel-based statistical analysis

Group-level analyses of GMV and ALFF were conducted using a general linear model framework implemented in *CAT12* and *CONN*, respectively. For each voxel, a separate model was estimated with voxel-wise GMV or ALFF as the dependent variable, and group, chronological age, sex, and total intracranial volume (GMV only) as independent variables. Statistical inferences were performed at the voxel level using familywise error correction for multiple comparisons, based on parametric statistics from Gaussian Random Field theory (Nieto-Castanon, 2020; Worsley et al., 1996). Results were thresholded using a combination of a cluster-forming voxel-level threshold (*P*_voxel_ < 0.001) and a familywise error-corrected cluster-size threshold (*P*_cluster_ < 0.05) (Chumbley, Worsley, Flandin, & Friston, 2010).

### Trait-phenotypes association analysis

To examine group differences and associations of interest, we applied general linear models adjusted for chronological age and sex. Given the substantial deviation from normality in cognitive performance measures, the Wilcoxon rank-sum test and Kendall’s tau correlation were employed. A significance threshold of *P*-value <0.05 was set for all statistical tests. For variables associated with transcriptomic aging, mediation and moderation analyses were further conducted to examine whether these variables mediated or moderated the relationship between transcriptomic aging and MDD, adjusting for age and sex as covariates (*bruceR* package). Effects were estimated using Markov Chain Monte Carlo simulations with 10,000 iterations.

MRI data processing was performed in *MATLAB and fMRIPrep*, polygenic risk scores were calculated using *PRSice-2*, and all remaining statistical analyses were conducted in *R.*

## Results

### Accelerated transcriptomic aging in MDD

**Figure 2.**
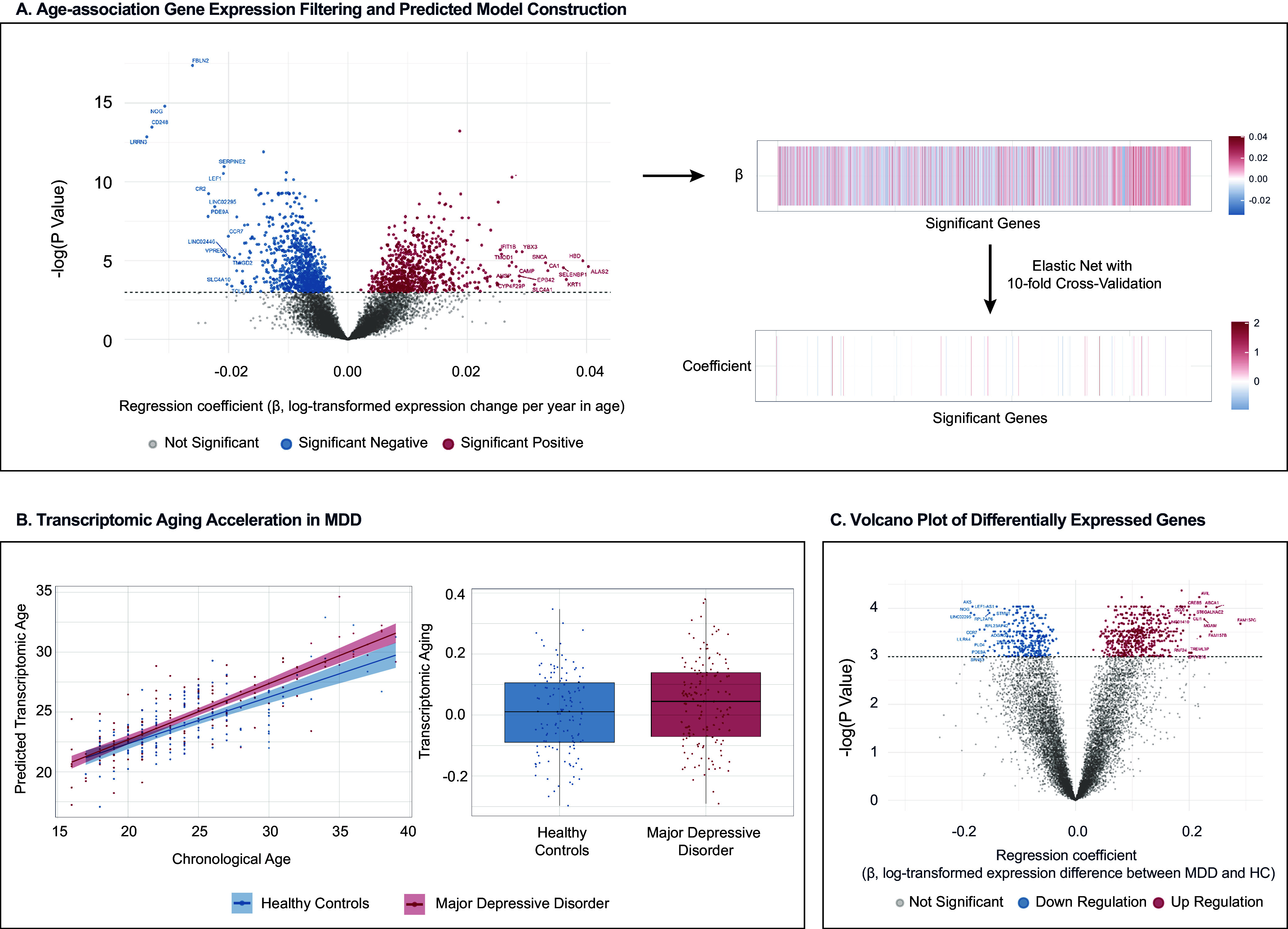
Age-association and MDD-association transcriptome analysis. *Note*: (a) Age-association gene expression filtering and predictive model construction. Genes associated with chronological age were identified using linear regression models, with filtering thresholds of P_FDR_ < 0.05 (left). The top 15 positive and top 15 negative genes are labeled in the figure. The significant genes are displayed (top right), followed by elastic net regression with 10-fold cross-validation to construct the transcriptomic age prediction model (bottom right), where selected genes and their model coefficients are shown. (b) Transcriptomic aging acceleration in MDD. The left plot shows the predicted transcriptomic age plotted against chronological age in HCs and MDD groups. The right boxplot compares transcriptomic aging acceleration between HCs and MDD groups. (c) Volcano plot of differentially expressed genes between MDD and HCs. The top 15 positive and top 15 negative genes are labeled in the figure. Abbreviations: MDD, ‘major depressive disorder’; HCs, ‘healthy controls’; FDR, ‘false discovery rate’. Figure 2. long description.

The final sample included 141 individuals with MDD and 134 HCs. The sex distribution did not differ significantly between groups (χ ^2^ < 0.001, *P* = 0.98), and no significant difference in chronological age was observed (*t* = 0.08, *P* = 0.94). A total of 1759 age-associated genes were identified, including 954 upregulated and 805 downregulated genes. To evaluate whether age-associated genes could predict chronological age, we trained a 10-fold cross-validated elastic-net model. This approach resulted in the selection of 55 genes, including 32 with negative coefficients and 23 with positive coefficients (Figure 2a). Participants in the MDD group exhibited significantly greater transcriptomic age acceleration than HCs after adjustment for chronological age and sex (*t* = 3.72, *P* < 0.001). This effect remained significant in the unadjusted model (*t* = 2.06, *P* = 0.04) (Figure 2b). In addition, we identified differently expressed genes between individuals with MDD and HCs, yielding a total of 837 genes, including 517 upregulated and 320 downregulated genes (Figure 2c).

### Gene ontology enrichment analysis

**Figure 3.**
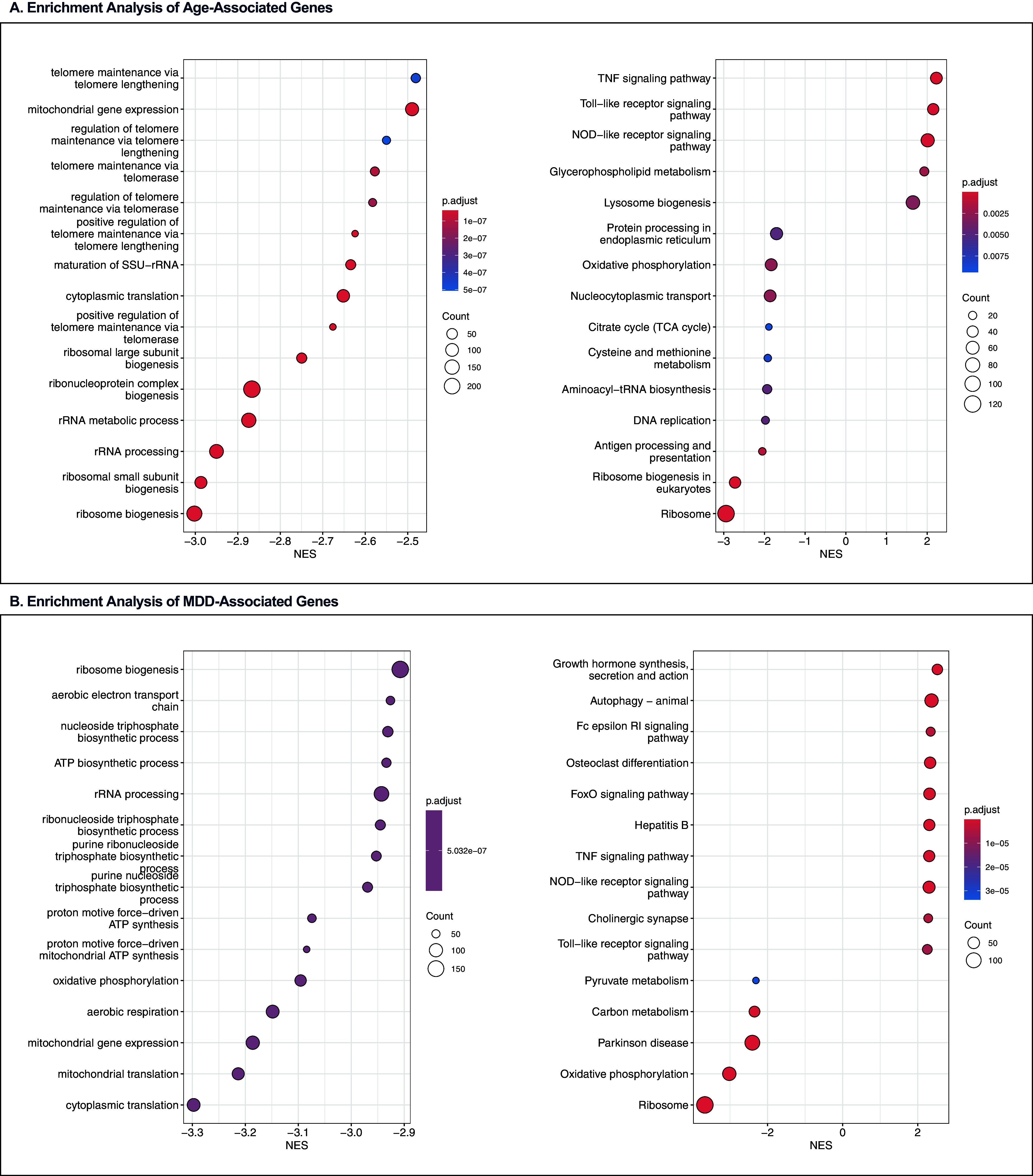
Gene ontology enrichment and network analysis of significant genes. *Note*: (a) Enrichment results for aging-associated genes. The left panel shows significantly enriched Gene Ontology biological process terms, and the right panel shows significantly enriched KEGG pathways. (b) Enrichment results for MDD-associated genes. The left panel shows significantly enriched Gene Ontology biological process terms, and the right panel shows significantly enriched KEGG pathways. Abbreviations: NES, ‘normalized enrichment score’. Figure 3. long description.

Age-associated genes were mainly enriched in pathways related to ribosome biogenesis, rRNA processing, telomere maintenance, and mitochondrial gene expression, with most GO terms showing negative NES values. KEGG analysis further highlighted downregulation of ribosome- and protein synthesis-related pathways, alongside positive enrichment of immune and inflammatory pathways such as Toll-like receptor, nucleotide-binding oligomerization domain-like receptor (NLR), and tumor necrosis factor (TNF) signaling (Figure 3a).

MDD-associated genes were mainly enriched in GO terms related to cytoplasmic translation, mitochondrial translation, oxidative phosphorylation, ATP biosynthesis, and ribosome biogenesis, with predominantly negative NES values. KEGG analysis further showed negative enrichment of ribosome and oxidative phosphorylation pathways, together with positive enrichment of immune-, stress-, and autophagy-related pathways, including Toll-like receptor, NOD-like receptor, TNF signaling, Fc epsilon RI signaling, and autophagy (Figure 3b).

#### Associations between transcriptomic aging and clinical phenotypes

No significant associations were observed between transcriptomic aging and clinical characteristics, including depression severity (*t* = 1.10, *P* = 0.27), anxiety symptoms (*t* = 0.16, *P* = 0.88), the number (*t* = −0.39, *P* = 0.70) or duration (*t* = 0.02, *P* = 0.98) of depressive episodes, hospitalizations due to depression (*t* = −0.47, *P* = 0.64), suicidal ideation (*t* = −0.36, *P* = 0.72), or suicidal behavior (*t* = 0.18, *P* = 0.86). In terms of neurocognitive performance, individuals with MDD exhibited significantly poorer performance compared to HCs across multiple domains, including visual memory, recognition memory, sustained attention, working memory, shifting, and planning. However, none of these cognitive measures showed significant correlations with transcriptomic aging (Table 1).

**Table 1.** Clinical characteristics and neurocognitive features of participants Table 1. long description.

Variables	Mean (SD) or Num (Percentage)		Group deviations		Associations with aging	
	Health controls (*N* = 134)	Major depressive disorder (*N* = 141)	Statistic	*P*-value	Statistic	*P*-value
Chronological age, y	24.4 (4.67)	24.4 (5.76)	*t =* 0.08	0.94	NA	NA
Sex, No. (%)						
Male	42 (31.3%)	43 (30.5%)	*χ ^2^* < 0.001	0.98	NA	NA
Female	92 (68.7%)	98 (69.5%)				
HDRS total score	NA	21.3 (4.72)	NA	NA	*t* = 1.10	0.27
HARS total score	NA	15.4 (5.95)	NA	NA	*t* = 0.16	0.88
Number of total depressive episodes	NA	1.78 (2.04)	NA	NA	*t* = −0.39	0.70
Duration of the current episode, mo	NA	7.18 (12.8)	NA	NA	*t* = −0.02	0.98
Number of hospitalizations due to depressive episodes	NA	0.393 (0.615)	NA	NA	*t* = −0.47	0.64
Suicidal ideation, No. (%)						
Yes	NA	98 (69.5%)	NA	NA	*t* = −0.36	0.72
No	NA	43 (30.5%)	NA			
Suicidal behavior, No. (%)						
Yes	NA	25 (17.7%)	NA	NA	*t* = 0.18	0.86
No	NA	116 (82.3%)	NA			
DMS_PC_A, %	89.9 (6.98)	82.8 (12.1)	*W* = 5012	<0.001	*Z* = 0.99	0.37
DMS_MCLA, s	3.68 (1.00)	3.67 (1.06)	*W* = 7723	0.46	*Z* = −1.55	0.12
PRM_PCd, %	84.9 (14.5)	74.9 (16.9)	*W* = 4706	<0.001	*Z* = −0.04	0.97
PRM_MCLd, s	1.97 (5.26)	1.98 (7.46)	*W* = 6887	0.71	*Z* = −1.21	0.23
RVP_PH, %	74.9 (16.5)	62.2 (19.6)	*W* = 4264	<0.001	*Z* = 0.06	0.95
RVP_ML, ms	380 (77.8)	419 (100)	*W* = 8886	<0.001	*Z* = −1.80	0.07
SWM_TE, No.	17.6 (17.5)	24.5 (17.2)	*W* = 9264	<0.001	*Z* = 0.57	0.57
SWM_Stra	31.2 (5.57)	33.2 (4.97)	*W* = 8690	0.004	*Z* = −0.76	0.45
IED_TE_A, No.	24.2 (27.9)	32.6 (31.3)	*W* = 9546	<0.001	*Z* = −0.46	0.65
IED_SC, No.	8.53 (1.09)	8.28 (1.36)	*W* = 6810	0.027	*Z* = 0.23	0.82
SOC_PS, No.	9.02 (1.72)	8.14 (2.13)	*W* = 5476	<0.001	*Z* = 1.49	0.14
SOC_MM5M, No.	6.36 (1.22)	6.84 (1.51)	*W* = 8518	0.009	*Z* = −1.78	0.07

*Note*: HDRS, Hamilton Depression Rating Scale; HARS, Hamilton Anxiety Rating Scale; DMS, delayed matching to sample; PRM, pattern recognition memory; RVP, rapid visual information processing; IED, intra–extra dimensional set shift; SOC, stockings of Cambridge; SWM, spatial working memory; MCL, mean correct latency; PC, percentage correct; PH, probability of hit; ML, mean latency; TE, total errors; Stra, strategy; SC, stage completed; PS, problems solved in minimum moves; MM, mean moves.

#### Associations between transcriptomic aging and environmental and biological risks

**Figure 4.**
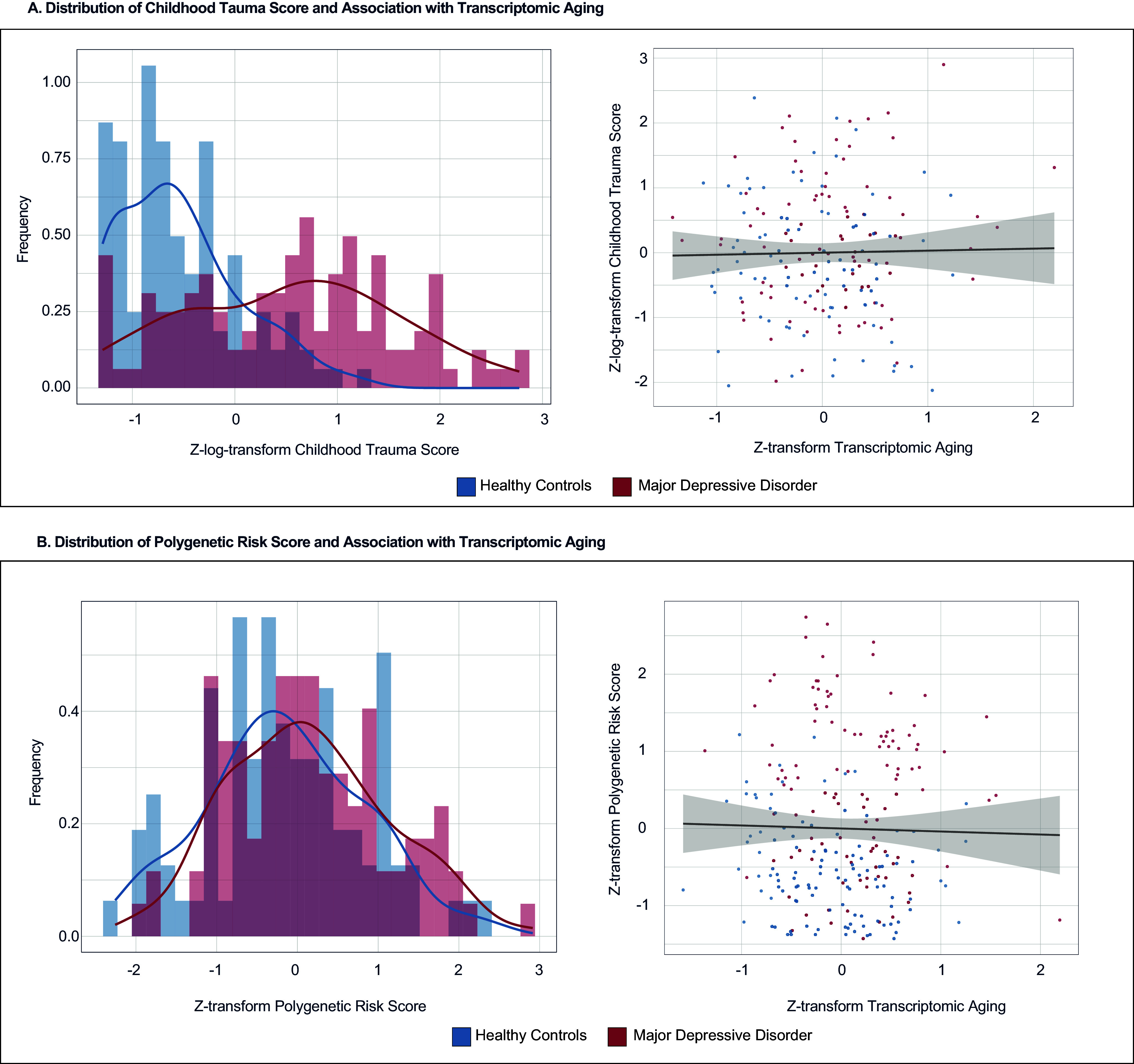
Distributions of risk factors and their associations with transcriptomic aging. *Note*: (a) Distribution of childhood trauma scores and their association with transcriptomic aging. The left histogram shows the distribution of childhood trauma scores for MDD and HCs, with density curves overlaid for each group. The right scatterplot illustrates the association between transcriptomic aging and childhood trauma scores, with a regression line and 95% confidence interval. (b) Distribution of PRS and their association with transcriptomic aging. The left histogram presents the distribution of PRS, and the right scatterplot displays the association with transcriptomic aging. Abbreviations: MDD, ‘major depressive disorder’; HCs, ‘healthy controls’; PRS, ‘polygenic risk score’. Figure 4. long description.

Childhood trauma scores were significantly higher in the MDD group compared to HCs (*t* = 9.69, *P* < 0.001) but were not significantly associated with transcriptomic aging (*t* = −0.34, *P* = 0.73) (Figure 4a). In addition, polygenic risk scores were significantly elevated in individuals with MDD relative to controls (*t* = 2.04, *P* = 0.046) but were not significantly associated with transcriptomic aging (*t* = 0.25, *P* = 0.80) (Figure 4b).

#### Associations between transcriptomic aging and neuroimages

**Figure 5.**
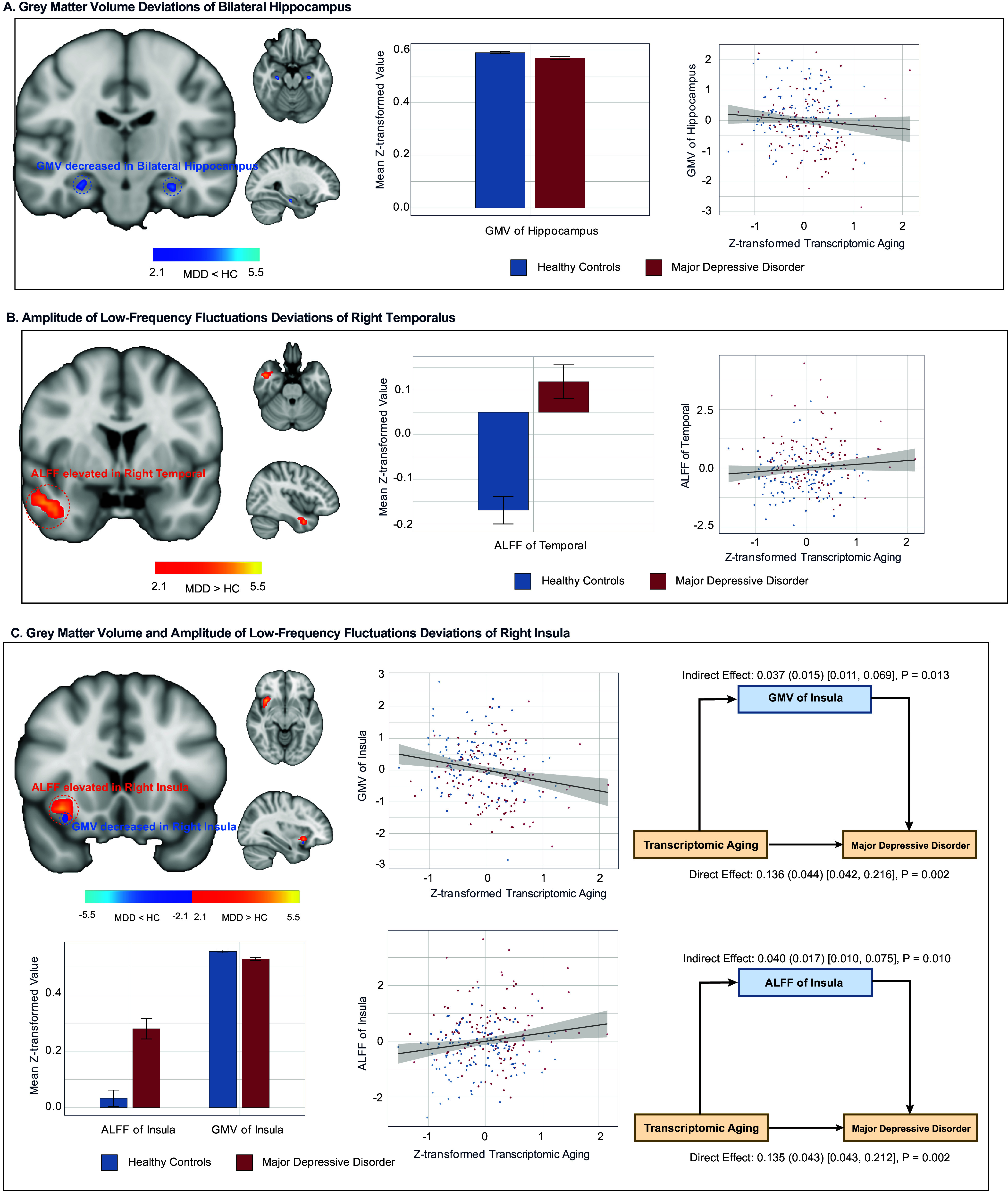
Brain structural and functional alterations associated with transcriptomic aging in MDD. *Note*: (a) GMV deviations in the bilateral hippocampus. The left image shows decreased GMV in the bilateral hippocampus in MDD compared to HCs. The middle bar plot presents the group comparison of mean GMV values. The right scatterplot shows the association between transcriptomic aging and hippocampal GMV. (b) ALFF deviations in the right temporal region. The left image displays elevated ALFF in the right temporal cortex in MDD. The middle bar plot shows the group difference in ALFF values. The right scatterplot depicts the association between transcriptomic aging and temporal ALFF. (c) GMV and ALFF deviations in the right insular cortex and mediation analyses. The left upper image demonstrates decreased GMV in the right insula in MDD compared to HCs, while the left lower bar plot presents the group comparison of mean GMV and ALFF values. The middle scatterplots illustrate the associations between transcriptomic aging and both GMV and ALFF in the right insula. The right diagrams present mediation models showing that both insular GMV and ALFF partially mediate the association between transcriptomic aging and MDD, with standardized indirect and direct effects indicated. Abbreviations: MDD, ‘major depressive disorder’; HCs, ‘healthy controls’; GMV, ‘gray matter volume’; ALFF, ‘amplitude of low-frequency fluctuation’. Figure 5. long description.

Among the final sample of 275 participants, 239 (121 MDD and 118 HCs) completed MRI scanning and passed image quality control. This subsample showed demographic characteristics (age: *t* = 0.032, *P* = 0.98; sex: χ ^2^ < 0.001, *P* = 0.998) and transcriptomic aging acceleration (uncorrected: *t* = 2.07, *P* = 0.04; corrected: *t* = 3.63, *P* < 0.001) largely comparable to those of the full sample. After voxel-level FWER correction, four significant clusters were identified. In the GMV analysis, one bilateral cluster was found with most voxels located in the hippocampal and parahippocampal regions (right: P_FWER_ = 0.020, size = 22, Montreal Neurological Institute (MNI) coordinates = 30, −18, −20; left: P_FWER_ = 0.024, size = 16, MNI coordinates = −28, −20, −20) (Figure 5a). Another GMV cluster was located in the right insula (P_FWER_ = 0.032, size = 10, MNI coordinates = 32, 16, −14) (Figure 5c). In the ALFF analysis, one cluster was primarily located in the right temporal pole and middle temporal gyrus (P_FWER_ = 0.038, size = 484, MNI coordinates = 46, 8, −26) (Figure 5b). Another cluster was identified with the majority of voxels in the right insula (P_FWER_ = 0.028, size = 204, MNI coordinates = 36, 14, −8) (Figure 5c).

Among the neuroimaging measures, transcriptomic aging was significantly associated with both reduced GMV (*t* = −3.30, *P* = 0.001) and increased ALFF (*t* = 2.64, *P* = 0.009) in the right insula (Figure 5c). No significant associations were found between transcriptomic aging and gray matter volume of the hippocampus (*t* = −1.39, *P* = 0.17) or ALFF in the temporal cortex (*t* = 1.44, *P* = 0.15) (Figure 5a,b). Mediation analysis revealed that both GMV and ALFF in the right insula significantly and partially mediated the association between transcriptomic aging and MDD (Figure 5c). No significant moderating effects of GMV or ALFF were observed in this relationship.

## Discussion

In the current study, we assessed biological aging in MDD based on peripheral transcriptomic markers and found evidence of accelerated aging. Similar patterns have been reported using other biological aging clocks, such as epigenetic and metabolomic clocks (Protsenko et al., 2021; Robinson et al., 2020). These observations suggest that accelerated biological aging may be linked to increased MDD risk even among individuals who are relatively young and have not yet reached the conventional definition of ‘old age’.

Previous studies have suggested that transcriptomic data exhibit substantial temporal and spatial variability, leading to challenges in developing a universally applicable transcriptomic aging clock (Rutledge, Oh, & Wyss-Coray, 2022). Nevertheless, the aim of this study was not to construct a generalized model, but to obtain a relatively robust estimate of transcriptomic age within our sample. Our results demonstrated a strong correlation between predicted transcriptomic age and chronological age, which compares favorably to previously reported correlations for peripheral blood (Peters et al., 2015) and brain transcriptomes (Zhao et al., 2022). This correlation remained lower than that of epigenomic (Han et al., 2018; Protsenko et al., 2021) or proteomic (Lehallier et al., 2019) clocks. This discrepancy may indicate the inherently weaker associations between transcriptomic profiles and chronological age. However, peripheral transcriptomic data retain informative value for capturing aging-related biological processes. A prior transcriptomic study based on peripheral mononuclear cells reported that individuals with MDD exhibited greater biological aging than HCs (Cole et al., 2021). Similarly, evidence from prefrontal cortex transcriptomic data has also shown accelerated biological aging in MDD and PTSD (Zhao et al., 2022). Our findings are consistent with these studies.

Chronological age represents an important source of systematic bias in the estimation of biological age. On one hand, aging reflects the cumulative effects of genomic profiles and environmental damage over time, and thus, the impact naturally becomes more pronounced with increasing chronological age (Moqri et al., 2023). On the other hand, regression models are subject to regression-to-the-mean effects, necessitating correction for age-related bias (Baecker et al., 2021). In the present study, we applied two strategies to minimize the influence of chronological age. First, we quantified the rate of transcriptomic aging by calculating the ratio of deviation to chronological age, whereas previous studies typically estimated biological aging based only on absolute deviation (Liu et al., 2024). Second, chronological age was included as a covariate in all subsequent statistical analyses. Our results showed that after adjusting for chronological age, the accelerated transcriptomic aging observed in individuals with MDD became more pronounced.

Our enrichment analysis in a Chinese sample similarly identified age-related pathways involving innate immune response, ribosome biogenesis, mitochondrial function, and telomere length, which is broadly consistent with findings from a large whole-blood transcriptomic study in predominantly European-ancestry populations (Peters et al., 2015). Building on this, we further found a partial overlap between MDD-associated genes and aging-associated genes, as both were positively associated with innate immune-related processes. Aging has been typically linked to chronic, sterile, low-grade systemic inflammation driven by innate immunity, a phenomenon known as ‘inflammaging’ (Franceschi et al., 2018; Franck et al., 2025). On the other hand, MDD has been increasingly associated with heightened innate immune-mediated inflammation (Beurel, Toups, & Nemeroff, 2020). In the periphery, this inflammatory dysregulation is reflected in altered levels of inflammatory mediators and shifts in immune cell composition (Foley et al., 2023; Zeng et al., 2024). In the central nervous system, it may manifest as microglial activation accompanied by changes in neuronal activity (Wang et al., 2022). Evidence from the central transcriptome in a US sample composed primarily of White participants suggests that genes linked to transcriptomic aging in MDD are mainly enriched in immune and inflammatory processes, in line with our findings (Zhao et al., 2022). Taken together, our findings suggest that innate immune-mediated inflammation may serve as a bridge linking aging and MDD.

Our results further suggest that, beyond inflammation, suppressed ribosome biogenesis may represent another overlapping mechanism linking aging and MDD. Impaired ribosome biogenesis can lead to the accumulation of unassembled free ribosomal proteins, which bind to and inhibit MDM2, a major negative regulator of p53, thereby increasing p53 activity and inducing cell-cycle arrest, senescence, or apoptosis (Cheng et al., 2024; Sirozh et al., 2024). This process, known as nucleolar stress, has been increasingly implicated in aging. Although nucleolar stress has not yet been established as a canonical mechanism of MDD, accumulating indirect evidence suggests that the nucleolus–ribosome axis disrupts in MDD and chronic stress, affecting ribosome biogenesis, rDNA transcription, and nucleolus-associated snoRNA regulation (Hori et al., 2018; Lin et al., 2024; Smagin et al., 2016; Zhang et al., 2023). Our findings raise the possibility that this shared disruption may represent a common biological pathway linking aging-related changes and MDD.

Our analyses further showed that both aging-associated genes and MDD-associated genes were enriched in mitochondrial energy metabolism-related pathways, particularly oxidative phosphorylation and mitochondrial gene expression. Previous studies have shown that aging is accompanied by the gradual accumulation of mtDNA mutations and progressive mitochondrial dysfunction, including oxidative phosphorylation defects, reduced membrane potential, increased reactive oxygen species, disturbed lipid metabolism, and imbalances in mitochondrial morphology and dynamics (Amorim et al., 2022; Xu, Pang, & Fan, 2025). In MDD, mitochondrial dysfunction has likewise been increasingly implicated in impaired energy metabolism, oxidative stress, and abnormal neuroplasticity (Jiang, Wang, & Sheng, 2024). Together, these findings support the possibility that aging and MDD may share disrupted mitochondrial energy metabolism as a common biological basis.

Furthermore, our results also identified processes that appeared to be more specifically related to transcriptomic aging. Aging was specifically associated with suppression of telomere length maintenance-related processes, whereas such enrichment was not observed for MDD. Given that telomere length is a well-established hallmark of aging, this finding is not unexpected. In contrast, the relationship between telomere length and MDD remains controversial. The previous study has reported shorter telomere length in patients with MDD, particularly in those with chronic or severe depression (Au Young, Teo, Parhar, & Soga, 2025). Stress-induced telomere damage may contribute to the linkage between chronic psychological stress and depression (Lin & Epel, 2022). However, our previous Mendelian randomization analysis did not support a causal relationship between telomere length and depression (Chen, Yan, Wang, & Xu, 2023). Taken together, these findings suggest that telomere shortening may reflect accumulated stress burden or disease chronicity rather than a core causal mechanism of MDD itself.

In the neuroimaging analyses, we found that transcriptomic aging was associated with structural and functional alterations in the right insular cortex, and that these alterations partially mediated the relationship between transcriptomic aging and MDD. The insula, a core hub of the salience network, plays a critical role in the generation and regulation of emotions by sensing and integrating changes in internal states (Etkin, Büchel, & Gross, 2015; Malezieux, Klein, & Gogolla, 2023). Recent perspectives suggest that the brain continuously monitors the activity of the immune system and modulates immune responses accordingly, with the insula serving as a key neural hub for neuro-immune interactions due to its integrative role in interoception and emotional processing (Rolls, 2023). Moreover, emerging evidence suggests that the insula may possess a form of ‘immune memory’, wherein immune events can leave specific neuronal activation patterns within the insular cortex (Rolls, 2023). Upon reactivation, these neuronal ensembles may elicit immune responses even after the initial inflammatory stimulus has resolved (Koren et al., 2021). Neuromodulation of the insular cortex may help restore immune balance, slow biological aging, and alleviate depressive symptoms.

The relationships between transcriptomic aging and clinical, neurocognitive, and early environmental and genetic phenotypes of MDD yielded negative findings. These results partially mirror a previous study based on epigenetic clocks, which found no associations between depressive symptoms and epigenetic aging but did observe associations with early life trauma (Han et al., 2018). Another study did not investigate the relationship between epigenetic aging and multidimensional phenotypes of MDD (Protsenko et al., 2021). While the heritability of MDD is around 30%, environmental factors are thought to have a greater impact (Bigdeli et al., 2017). However, neither genetics nor environment alone can fully explain the pathogenesis of MDD. Transcriptomic changes in MDD are more likely a trait marker shaped by long-term gene – environment interactions, rather than a transient state driven by single genetic or environmental factors. These observations raise the possibility that accelerated transcriptomic aging represents an integrative indicator of MDD vulnerability.

The study has several limitations. First, although efforts were made to minimize medication effects and maintain sample homogeneity, this was still a single-center case–control study with a relatively small sample size and no longitudinal follow-up, which may limit the generalizability of the findings and preclude causal inferences. Second, because gene selection and model construction were performed in the same sample, age prediction may be optimistic, and transcriptomic aging may be underestimated. Although group comparisons are unlikely to be substantially affected, generalizability may still be limited by potential overfitting. Third, some potentially relevant covariates, such as smoking status and body mass index, were not included in the present analysis and may have influenced peripheral transcriptomic profiles. Finally, our analysis was limited to mRNA expression and did not include other transcriptomic components such as ribosomal RNA, microRNAs, and long noncoding RNAs.

## Conclusion

In conclusion, accelerated transcriptomic aging is associated with an increased risk of MDD, and innate immune inflammation, ribosome biogenesis, and mitochondrial energy metabolism may represent potential mechanisms underlying this association. The structural and functional abnormalities of the insular cortex may indicate altered interoception, suggesting that the insula may be one of the regions affected by aging-related alterations. These findings provide potential insight for understanding how aging-related biological alterations may contribute to the pathophysiology of MDD.

## Supporting information

10.1017/S003329172610498X.sm001 Yan et al. supplementary material Yan et al. supplementary material

## Supplementary material

The supplementary material for this article can be found at http://doi.org/10.1017/S003329172610498X.

## Long descriptions

### Figure 1. Long description

The flowchart is organized into two horizontal stages.

Top Row: Transcriptomic Aging Estimation

1. Peripheral Blood Collection and Transcriptome Sequencing: An illustration shows a needle drawing blood from an arm into a tube, leading to a red D N A double helix icon.

2. Linear Model: An arrow labeled Linear Model points to a group of blue R N A strand icons labeled Age-related Genes Expression.

3. Elastic Net Model: An arrow labeled Elastic Net Model points to a scatter plot titled Transcriptomic Aging Estimation. The plot features Actual Age on the x-axis and Predicted Age on the y-axis, with purple data points and a diagonal line of best fit.

Bottom Row: Comparative and Phenotype Analysis

1. Group-Deviation Analysis: On the far left, three blue icons represent Major Depressive Disorder and three orange icons represent Health Controls. An arrow points from these groups toward a central dashed box.

2. Trait-Phenotype Analysis: An L-shaped arrow descends from the top-right scatter plot and points leftward into the same central dashed box.

3. Central Analysis Box: This box contains seven icons representing the variables analyzed:

- Differential Expressed Genes (R N A strands)

- Polygenic Risk Score (D N A helix)

- Clinical Symptoms (person with a clipboard)

- Neurocognitive Function (brain icon)

- Childhood Trauma (person in a fetal position)

- Grey Matter Volume (anatomical brain cross-section)

- Amplitude of Low Frequency Fluctuations (brain cross-section with red heat map highlights).

Navigate back to Figure 1..

### Figure 2. Long description

Panel A: Age-association Gene Expression Filtering. A volcano plot on the left shows minus log P Value on the y-axis and regression coefficient beta on the x-axis. Blue dots represent significant negative genes (e.g., F B L N 2, N O G, C D 2 4 8) and red dots represent significant positive genes (e.g., H B Q, A L A S 2, K R T 1). To the right, a flow diagram shows these significant genes entering an Elastic Net with 10-fold Cross-Validation, resulting in a coefficient heatmap where specific genes are selected for the predictive model.

Panel B: Transcriptomic Aging Acceleration in M D D. The left scatter plot shows Predicted Transcriptomic Age (y-axis) versus Chronological Age (x-axis). Both Healthy Controls (blue) and Major Depressive Disorder (red) groups show a linear increase, but the red M D D line is positioned higher. The right boxplot confirms this, showing higher Transcriptomic Aging values for the Major Depressive Disorder group compared to Healthy Controls.

Panel C: Volcano Plot of Differentially Expressed Genes. This plot compares M D D and H C s. The y-axis is minus log P Value and the x-axis is the regression coefficient. Blue dots indicate Down Regulation and red dots indicate Up Regulation. Top labeled up-regulated genes include A V P, C R E B 3 L 2, and A B C A 1, while down-regulated labels include L I N C 0 2 3 8 1 and P T G E R 4.

Navigate back to Figure 2..

### Figure 3. Long description

The figure consists of two main sections, A and B, each containing two dot plots.

Section A: Enrichment Analysis of Age-Associated Genes.

* Left Plot (Gene Ontology): The x-axis shows N E S from minus 3.0 to minus 2.5. Terms include ribosome biogenesis, r R N A processing, and mitochondrial gene expression. Larger red dots (higher gene count, lower p-value) are concentrated at the bottom left, indicating strong negative enrichment for ribosome-related processes.

* Right Plot (K E G G Pathways): The x-axis ranges from minus 3 to plus 2. Positive enrichment (N E S 1 to 2) is seen for T N F signaling, Toll-like receptor signaling, and Lysosome. Negative enrichment (N E S minus 3 to minus 1) is seen for Ribosome and Oxidative phosphorylation.

Section B: Enrichment Analysis of M D D-Associated Genes.

* Left Plot (Gene Ontology): The x-axis shows N E S from minus 3.3 to minus 2.9. Top terms include ribosome biogenesis and aerobic electron transport chain. The largest dots are at the top and bottom of the list, showing negative enrichment for translation and metabolic processes.

* Right Plot (K E G G Pathways): The x-axis ranges from minus 3 to plus 2. Positive enrichment is shown for Growth hormone synthesis, FoxO signaling, and T N F signaling. Negative enrichment is shown for Ribosome, Parkinson disease, and Oxidative phosphorylation.

Legends for all plots indicate that dot size represents ‘Count’ (ranging from 20 to 200) and dot color represents ‘p dot adjust’ (ranging from blue for higher values to red for lower values like 1e minus 07).

Navigate back to Figure 3..

### Table 1. Long description

The table is organized into seven columns: Variables, Mean (S D) or Num (Percentage) for Health controls (N = 134) and Major depressive disorder (N = 141), Group deviations (Statistic and P-value), and Associations with aging (Statistic and P-value).

* Chronological age: Both groups mean 24.4 years. Group deviation t = 0.08, P = 0.94.

* Sex: Health controls are 31.3% male and 68.7% female; M D D group is 30.5% male and 69.5% female. Chi-squared < 0.001, P = 0.98.

* Clinical Scores (M D D only): H D R S total score 21.3; H A R S total score 15.4; Total depressive episodes 1.78; Current episode duration 7.18 months; Hospitalizations 0.393. Suicide ideation is present in 69.5% and attempts in 17.7%.

* Neurocognitive Features (Health vs M D D):

- D M S _ P C _ A: 89.9% vs 82.8% (P < 0.001).

- D M S _ M C L A: 3.68 s vs 3.67 s (P = 0.46).

- P R M _ P C d: 84.9% vs 74.9% (P < 0.001).

- R V P _ P H: 74.9% vs 62.2% (P < 0.001).

- R V P _ M L: 380 ms vs 419 ms (P < 0.001).

- S W M _ T E: 17.6 vs 24.5 (P < 0.001).

- I E D _ T E _ A: 24.2 vs 32.6 (P < 0.001).

- S O C _ P S: 9.02 vs 8.14 (P < 0.001).

Associations with aging statistics (Z-values) range from -1.80 to 1.49, with all P-values > 0.05, indicating no significant correlation with age in this sample.

Navigate back to Table 1..

### Figure 4. Long description

Panel A, top row. The left histogram shows the distribution of Z-log-transform Childhood Trauma Score on the X-axis against Frequency on the Y-axis. Healthy Controls (blue) are concentrated on the left (lower scores), while Major Depressive Disorder (red) shows a broader, higher distribution. The right scatterplot shows Z-transform Transcriptomic Aging on the X-axis and Z-log-transform Childhood Trauma Score on the Y-axis. A near-horizontal regression line with a grey 95% confidence interval indicates a weak or negligible correlation.

Panel B, bottom row. The left histogram shows the distribution of Z-transform Polygenic Risk Score on the X-axis against Frequency on the Y-axis. Both Healthy Controls (blue) and Major Depressive Disorder (red) show overlapping bell-shaped distributions centered near zero. The right scatterplot shows Z-transform Transcriptomic Aging on the X-axis and Z-transform Polygenic Risk Score on the Y-axis. The regression line is slightly negative but nearly flat, with a widening 95% confidence interval at the extremes, suggesting no significant association.

Navigate back to Figure 4..

### Figure 5. Long description

A multi-panel figure organized into three horizontal sections labeled A, B, and C.

Panel A: Grey Matter Volume (G M V) Deviations of Bilateral Hippocampus.

- Left: Three brain scan views (coronal, axial, sagittal) show blue-shaded regions indicating decreased G M V in the bilateral hippocampus for M D D compared to H C s. A color scale ranges from 2.1 to 5.5 for M D D less than H C.

- Middle: A bar plot of Mean Z-transformed Value shows H C s (blue bar) slightly higher than M D D (red bar) for G M V of Hippocampus.

- Right: A scatterplot shows a negative linear correlation between Z-transformed Transcriptomic Aging (x-axis) and G M V of Hippocampus (y-axis).

Panel B: Amplitude of Low-Frequency Fluctuations (A L F F) Deviations of Right Temporalus.

- Left: Brain scans show orange-red regions indicating elevated A L F F in the right temporal cortex. A color scale ranges from 2.1 to 5.5 for M D D greater than H C.

- Middle: A bar plot shows H C s (blue bar) with a negative mean value and M D D (red bar) with a positive mean value for A L F F of Temporal.

- Right: A scatterplot shows a slight positive linear correlation between Transcriptomic Aging and A L F F of Temporal.

Panel C: G M V and A L F F Deviations of Right Insula.

- Top Left: Brain scans show overlapping regions of decreased G M V (blue) and elevated A L F F (orange) in the right insula.

- Bottom Left: A bar plot compares A L F F of Insula (M D D higher than H C) and G M V of Insula (H C higher than M D D).

- Center: Two scatterplots show a negative correlation for G M V of Insula and a positive correlation for A L F F of Insula against Transcriptomic Aging.

- Right: Two mediation diagrams. The top model shows G M V of Insula partially mediating the path from Transcriptomic Aging to M D D (Indirect Effect 0.037, P equals 0.013; Direct Effect 0.136, P equals 0.002). The bottom model shows A L F F of Insula as a mediator (Indirect Effect 0.040, P equals 0.010; Direct Effect 0.135, P equals 0.002).

Navigate back to Figure 5..
