# Supplementary material for: Peripheral transcriptomic aging acceleration in major depressive disorder: the mediating role of insular cortex alterations
Source: Psychol Med. 2026 Jul 8;56:e213. doi: 10.1017/S003329172610498X (PMC13370190; doi:10.1017/S003329172610498X)
Supplement: Yan et al. supplementary material [file S003329172610498Xsup001.docx]

**Supplementary Method 1 Process of Peripheral RNA Extraction and Sequencing**

Venous blood was drawn and collected into Tempus Blood RNA Tubes (Applied Biosystems, Foster City, CA) for RNA stabilization. Tubes were vigorously inverted for at least 10 seconds to ensure thorough mixing of the blood with the stabilizing reagent and to prevent RNA degradation. Samples were then stored at –80 °C until further processing.

Total RNA was extracted using the MagMAX for Stabilized Blood Tubes RNA Isolation Kit (Thermo Fisher Scientific, Waltham, MA, USA) following the manufacturer’s protocol. RNA concentration and purity were assessed using a NanoDrop ND-2000 spectrophotometer by measuring absorbance at 260 nm and calculating the OD260/280 and OD260/230 ratios. RNA integrity evaluation and sequencing were performed by GENEWIZ Suzhou (Suzhou Genewiz Biotechnology Co., Ltd., Suzhou, China). RNA quality was assessed using an Agilent Bioanalyzer, and only samples with an RNA Integrity Number (RIN) > 6.5 were included for library preparation and subsequent high-throughput sequencing.

For transcriptomic sequencing, 1 μg of total RNA per sample was used for library preparation. The mRNA was enriched using oligo (dT) magnetic beads to remove most rRNA and globin-related non-mRNA signals and then fragmented into short segments ranging from approximately 200 to 500 nucleotides. First-strand cDNA synthesis was performed using reverse transcriptase, followed by second-strand synthesis with DNA polymerase I. After purification, the double-stranded cDNA underwent end repair and A-tailing before ligation with Illumina-compatible sequencing adapters. The ligated products were amplified by PCR to generate sequencing libraries.

RNA sequencing was performed using a NovaSeq 6000 Sequencing System (Illumina) with a 2 ×150 paired-end (PE) configuration, following the manufacturer’s guidelines. Raw sequencing reads were processed using Cutadapt (v1.9.1) to remove low-quality bases and adapter sequences, resulting in a set of high-quality clean reads that were subsequently aligned to the human reference genome using Hisat2 (v2.0.1).

**Supplementary Method 2 Process of Genomic DNA Extraction and Sequencing**

Genomic DNA was extracted from peripheral blood cells using the phenol-chloroform protocol. Briefly, 4 ml of whole blood was mixed with 10 ml of lysis buffer (1 M Tris·Cl, pH 7.0; 1 M MgCl₂; 10% sucrose; and 1% Triton X-100) and centrifuged at 3000 rpm for 20 minutes to remove red blood cells. The supernatant was discarded, and 2.5 ml STE buffer containing 250 μl 10% SDS and 25 μl proteinase K was added. The mixture was incubated at 37 °C for 4 hours to facilitate protein digestion. DNA was then extracted sequentially with phenol, phenol/chloroform (1:1), and chloroform/isoamyl alcohol (24:1). The supernatant was transferred to a fresh tube and DNA was precipitated by adding 2 volumes of cold ethanol and 1/10 volume sodium acetate (3M). The resulting DNA pellet was washed with 70% ethanol, air-dried, and dissolved in TE buffer. Samples were stored at –20 °C until further use. DNA concentration was measured by absorbance at 260 nm, and purity was assessed by the OD 260/280 ratio.

Genomic DNA samples were submitted to Meiji Gene (Shanghai Biowing Applied Biotechnology Co., Ltd., Shanghai, China) for genotyping. Genotyping was performed using the Illumina BeadChip Array Global Screening Array-24+ v1.0 platform according to the manufacturer’s standard protocol.

Genotype quality control was performed using PLINK (v1.9). First, SNPs with a minor allele frequency (MAF) < 0.05, Hardy-Weinberg equilibrium (HWE) p-value < 1×10⁻⁵, SNP call rate < 95%, or individual-level missingness > 5% were excluded. Linkage disequilibrium (LD) pruning was then conducted using a sliding window of 200 SNPs, advancing 50 SNPs at a time, and removing SNPs with pairwise LD r² > 0.25. Next, individuals with excessive heterozygosity (±5 standard deviations from the mean) or sex discrepancies were excluded. Ambiguous, mismatching, and duplicate SNPs were also removed. For related individuals with a pairwise identity-by-descent (PI-HAT) > 0.125, one member of each pair was excluded to ensure unrelatedness. After all quality control steps, 242,681 variants remained for downstream analysis.

**Supplementary Method 3 T1-weighted Structural MRI Preprocessing Procedure**

First, images underwent spatial adaptive non-local means (SANLM) denoising to reduce image noise while preserving anatomical details ^1^. The denoised images were then internally resampled to account for differences in resolution and voxel size, followed by bias-field correction and affine registration. Unified segmentation was subsequently applied to provide initial tissue classification into gray matter (GM), white matter (WM), and cerebrospinal fluid (CSF) ^2^. Refined voxel-based processing then proceeded with skull stripping and anatomical parcellation into the left and right hemispheres, subcortical structures, and cerebellum. Local white matter hyperintensities were detected and marked to improve spatial normalization and cortical thickness estimation. A local intensity transformation was performed to reduce regional intensity variability, particularly in motor and occipital cortices and basal ganglia. This was followed by adaptive maximum a posteriori (AMAP) segmentation, which does not rely on tissue priors and is refined using partial volume estimation^3,4^. Finally, tissue segments were spatially normalized to MNI space using the Geodesic Shooting algorithm ^5^. Modulated GM images were smoothed using an 8 mm full-width at half-maximum (FWHM) Gaussian kernel. Total intracranial volume (TIV) was computed for each participant and used as a covariate in group-level analyses where appropriate.

**Supplementary Method 4 Rest-state Functional MRI Preprocessing Procedure**

1. **Anatomical data preprocessing**

The T1-weighted (T1w) image was corrected for intensity non-uniformity (INU) with *N4BiasFieldCorrection*^6^, distributed with ANTs^7^, and used as T1w-reference throughout the workflow. The T1w-reference was then skull-stripped with a *Nipype* implementation of the *antsBrainExtraction.sh* workflow (from ANTs), using *OASIS30ANTs* as target template. Brain tissue segmentation of cerebrospinal fluid (CSF), white-matter (WM) and gray-matter (GM) was performed on the brain-extracted T1w using *fast*^8^ (FSL). Volume-based spatial normalization to one standard space (*MNI152NLin2009cAsym*) was performed through nonlinear registration with *antsRegistration* (ANTs), using brain-extracted versions of both T1w reference and the T1w template. The following template was were selected for spatial normalization and accessed with *TemplateFlow*^9^: *ICBM 152 Nonlinear Asymmetrical template version 2009c*^10^.

1. **Functional data preprocessing**

First, a reference volume was generated, using a custom methodology of *fMRIPrep*, for use in head motion correction. Head-motion parameters with respect to the BOLD reference (transformation matrices, and six corresponding rotation and translation parameters) are estimated before any spatiotemporal filtering using *mcflirt*^11^ (FSL). The BOLD reference was then co-registered to the T1w reference using *mri_coreg* (FreeSurfer) followed by *flirt*^12^ (FSL) with the boundary-based registration^13^ cost-function. Co-registration was configured with six degrees of freedom. Several confounding time-series were calculated based on the *preprocessed BOLD*: framewise displacement (FD), DVARS and three region-wise global signals. FD was computed using two formulations following Power^14^ (absolute sum of relative motions) and Jenkinson^11^ (relative root mean square displacement between affines). FD and DVARS are calculated for each functional run, both using their implementations in *Nipype* (following the definitions by Power et al. 2014). The three global signals are extracted within the CSF, the WM, and the whole-brain masks. Additionally, a set of physiological regressors were extracted to allow for component-based noise correction^15^ (*CompCor*). Principal components are estimated after high-pass filtering the *preprocessed BOLD* time-series (using a discrete cosine filter with 128s cut-off) for the two *CompCor* variants: temporal (tCompCor) and anatomical (aCompCor). tCompCor components are then calculated from the top 2% variable voxels within the brain mask. For aCompCor, three probabilistic masks (CSF, WM and combined CSF+WM) are generated in anatomical space. The implementation differs from that of Behzadi et al. in that instead of eroding the masks by 2 pixels on BOLD space, a mask of pixels that likely contain a volume fraction of GM is subtracted from the aCompCor masks. This mask is obtained by thresholding the corresponding partial volume map at 0.05, and it ensures components are not extracted from voxels containing a minimal fraction of GM. Finally, these masks are resampled into BOLD space and binarized by thresholding at 0.99 (as in the original implementation). Components are also calculated separately within the WM and CSF masks. For each CompCor decomposition, the *k* components with the largest singular values are retained, such that the retained components’ time series are sufficient to explain 50 percent of variance across the nuisance mask (CSF, WM, combined, or temporal). The remaining components are dropped from consideration. The head-motion estimates calculated in the correction step were also placed within the corresponding confounds file. The confound time series derived from head motion estimates and global signals were expanded with the inclusion of temporal derivatives and quadratic terms for each^16^. Frames that exceeded a threshold of 0.5 mm FD or 1.5 standardized DVARS were annotated as motion outliers. Additional nuisance timeseries are calculated by means of principal components analysis of the signal found within a thin band (*crown*) of voxels around the edge of the brain, as proposed by Patriat et al.^17^ All resamplings can be performed with *a single interpolation step* by composing all the pertinent transformations (i.e. head-motion transform matrices, susceptibility distortion correction when available, and co-registrations to anatomical and output spaces). Gridded (volumetric) resamplings were performed using *nitransforms*, configured with cubic B-spline interpolation. Functional data were smoothed using spatial convolution with a Gaussian kernel of 8 mm full width half maximum (FWHM).

1. **Denoising**

In addition, functional data were denoised using a standard denoising pipeline^18^ including the regression of potential confounding effects characterized by white matter timeseries (5 CompCor noise components), CSF timeseries (5 CompCor noise components), motion parameters and their first order derivatives (12 factors)^19^, session and task effects and their first order derivatives (2 factors), and linear trends (2 factors) within each functional run, followed by bandpass frequency filtering of the BOLD timeseries^20^ between 0.01 Hz and 0.1 Hz. CompCor^15,21^ noise components within white matter and CSF were estimated by computing the average BOLD signal as well as the largest principal components orthogonal to the BOLD average, motion parameters within each subject's eroded segmentation masks.

1. **First-level analysis**

Amplitude of low frequency fluctuations (ALFF) maps characterizing low-frequency BOLD signal variability at each voxel were estimated as the root mean square (RMS) of the BOLD signal after denoising and band-pass filtering between 0.01 Hz and 0.1 Hz^22^. ALFF measures across voxels were then rank sorted and normalized separately for each individual subject using a Gaussian inverse cumulative distribution function with zero mean and unit variance.

**Reference**

1. Manjón JV, Coupé P, Martí‐Bonmatí L, Collins DL, Robles M. Adaptive non‐local means denoising of MR images with spatially varying noise levels. *Magnetic Resonance Imaging*. 2010;31(1):192-203. doi:10.1002/jmri.22003

2. Ashburner J, Friston KJ. Unified segmentation. *NeuroImage*. 2005;26(3):839-851. doi:10.1016/j.neuroimage.2005.02.018

3. Rajapakse JC, Giedd JN, Rapoport JL. Statistical approach to segmentation of single-channel cerebral MR images. *IEEE Trans Med Imaging*. 1997;16(2):176-186. doi:10.1109/42.563663

4. Tohka J, Zijdenbos A, Evans A. Fast and robust parameter estimation for statistical partial volume models in brain MRI. *NeuroImage*. 2004;23(1):84-97. doi:10.1016/j.neuroimage.2004.05.007

5. Ashburner J, Friston KJ. Diffeomorphic registration using geodesic shooting and Gauss–Newton optimisation. *NeuroImage*. 2011;55(3):954-967. doi:10.1016/j.neuroimage.2010.12.049

6. Tustison NJ, Avants BB, Cook PA, et al. N4ITK: Improved N3 Bias Correction. *IEEE Trans Med Imaging*. 2010;29(6):1310-1320. doi:10.1109/TMI.2010.2046908

7. Avants B, Epstein C, Grossman M, Gee J. Symmetric diffeomorphic image registration with cross-correlation: Evaluating automated labeling of elderly and neurodegenerative brain. *Medical Image Analysis*. 2008;12(1):26-41. doi:10.1016/j.media.2007.06.004

8. Zhang Y, Brady M, Smith S. Segmentation of brain MR images through a hidden Markov random field model and the expectation-maximization algorithm. *IEEE Trans Med Imaging*. 2001;20(1):45-57. doi:10.1109/42.906424

9. Ciric R, Thompson WH, Lorenz R, et al. TemplateFlow: FAIR-sharing of multi-scale, multi-species brain models. *Nat Methods*. 2022;19(12):1568-1571. doi:10.1038/s41592-022-01681-2

10. Fonov V, Evans A, McKinstry R, Almli C, Collins D. Unbiased nonlinear average age-appropriate brain templates from birth to adulthood. *NeuroImage*. 2009;47(Supplement 1):S102. doi:10.1016/S1053-8119(09)70884-5

11. Jenkinson M, Bannister P, Brady M, Smith S. Improved Optimization for the Robust and Accurate Linear Registration and Motion Correction of Brain Images. *NeuroImage*. 2002;17(2):825-841. doi:10.1006/nimg.2002.1132

12. Jenkinson M, Smith S. A global optimisation method for robust affine registration of brain images. *Medical Image Analysis*. 2001;5(2):143-156. doi:10.1016/S1361-8415(01)00036-6

13. Greve DN, Fischl B. Accurate and robust brain image alignment using boundary-based registration. *NeuroImage*. 2009;48(1):63-72. doi:10.1016/j.neuroimage.2009.06.060

14. Power JD, Mitra A, Laumann TO, Snyder AZ, Schlaggar BL, Petersen SE. Methods to detect, characterize, and remove motion artifact in resting state fMRI. *NeuroImage*. 2014;84:320-341. doi:10.1016/j.neuroimage.2013.08.048

15. Behzadi Y, Restom K, Liau J, Liu TT. A component based noise correction method (CompCor) for BOLD and perfusion based fMRI. *NeuroImage*. 2007;37(1):90-101. doi:10.1016/j.neuroimage.2007.04.042

16. Satterthwaite TD, Elliott MA, Gerraty RT, et al. An improved framework for confound regression and filtering for control of motion artifact in the preprocessing of resting-state functional connectivity data. *NeuroImage*. 2013;64:240-256. doi:10.1016/j.neuroimage.2012.08.052

17. Patriat R, Reynolds RC, Birn RM. An improved model of motion-related signal changes in fMRI. *NeuroImage*. 2017;144:74-82. doi:10.1016/j.neuroimage.2016.08.051

18. Nieto-Castanon A. *Handbook of Functional Connectivity Magnetic Resonance Imaging Methods in CONN*. Hilbert Press; 2020. doi:10.56441/hilbertpress.2207.6598

19. Friston KJ, Williams S, Howard R, Frackowiak RSJ, Turner R. Movement‐Related effects in fMRI time‐series. *Magnetic Resonance in Med*. 1996;35(3):346-355. doi:10.1002/mrm.1910350312

20. Hallquist MN, Hwang K, Luna B. The nuisance of nuisance regression: Spectral misspecification in a common approach to resting-state fMRI preprocessing reintroduces noise and obscures functional connectivity. *NeuroImage*. 2013;82:208-225. doi:10.1016/j.neuroimage.2013.05.116

21. Chai XJ, Castañón AN, Öngür D, Whitfield-Gabrieli S. Anticorrelations in resting state networks without global signal regression. *NeuroImage*. 2012;59(2):1420-1428. doi:10.1016/j.neuroimage.2011.08.048

22. Yang H, Long XY, Yang Y, et al. Amplitude of low frequency fluctuation within visual areas revealed by resting-state functional MRI. *NeuroImage*. 2007;36(1):144-152. doi:10.1016/j.neuroimage.2007.01.054
